# Validity and test–retest reliability of the Swedish version of the Geriatric Depression Scale among very old adults

**DOI:** 10.1186/s12877-024-04869-7

**Published:** 2024-03-18

**Authors:** Sandra Snellman, Carl Hörnsten, Birgitta Olofsson, Yngve Gustafson, Hugo Lövheim, Johan Niklasson

**Affiliations:** 1 Department of Community Medicine and Rehabilitation, Geriatric Medicine, Sunderby Research Unit, Umeå University, Umeå, 901 87 Sweden; 2 Department of Community Medicine and Rehabilitation, Geriatric Medicine, Umeå University, Umeå, Sweden; 3 Department of Nursing, Umeå University, Umeå, Sweden; 4 Department of Clinical Sciences, Psychiatry, Umeå University, Umeå, Sweden; 5 Department of Diagnostics and Intervention, Orthopedics, Umeå University, Umeå, Sweden

**Keywords:** Aged 80 and over, Depression, Psychometrics, ROC curve, Psychiatric status rating scales

## Abstract

**Background:**

The Geriatric Depression Scale (GDS) has shown good validity and reliability, but few studies have examined the GDS among very old adults or the Swedish translation.

**Objectives:**

Evaluate the validity and reliability of the Swedish version of GDS-15 among very old adults.

**Methods:**

In the Umeå85 + /GErontological Regional DAtabase (GERDA) study, 387 participants were assessed with both the GDS-15 and the Montgomery-Åsberg Depression Rating Scale (MADRS). The mean age was 91 years. Concurrent validity between the scales was calculated using Spearman's correlation. We used the Diagnostic and Statistical Manual of Mental Disorders (DSM) V symptom criteria for depression based on MADRS item scores to define depression. We calculated the Area Under the Curve (AUC) and found an optimal cut-off.

A convenience sample with 60 individuals was used to calculate test–retest reliability with Cohen’s kappa and Intraclass Correlation Coefficient (ICC).

**Results:**

Spearman's correlation coefficients between total scores for GDS-15 and MADRS were 0.60. Cronbach's alpha for the whole scale was 0.73. The AUC was 0.90 for distinguishing major depression, and the recommended cut-off of ≥ 5 showed a sensitivity of 95.2% and specificity of 65.8%. The test–retest showed that Cohen’s kappa was substantial (0.71) and the ICC was excellent (0.95).

**Conclusions:**

The Swedish version of the GDS-15 showed good validity and reliability among very old adults. The generally recommended cut-off of ≥ 5 seems reasonable to use with the Swedish version and among very old adults.

**Supplementary Information:**

The online version contains supplementary material available at 10.1186/s12877-024-04869-7.

## Introduction

Globally, the percentage of older adults is increasing. The United Nations (UN) estimates that by 2060, the number of older people will double from approximately 9% to approximately 18% [1]. In a systematic review article, Luppa and coworkers found that the pooled prevalence of depressive disorders according to assessment scales among those aged 75 or older was 17.1% (95% confidence interval 9.7–26.1%). Moreover, the rates of depression increase substantially among people in the age group 85–89 by 20–25% and 90 years and older by 30–50% [2]. Similar results were found in a Swedish study among very old adults, where the prevalence of depression in 85-year-olds was 16.8% and increased among 90-year-olds and 95-year-olds and older to 34.1% and 32.3%, respectively. In addition to the high prevalence, undertreatment of depression was found where 33% had no antidepressant treatment and 59% were still depressed despite antidepressant treatment [3].

The Geriatric Depression Scale-15 item version (GDS-15) was created in English in 1986 from the GDS-30 item version [4]. It has been translated into more than 30 languages and is one of the most commonly used depression screening tools administered worldwide in geriatric populations. The questionnaire was designed to be answered with a simple "yes" or "no" answer, facilitating ease of use for older individuals, including those with impaired cognition. Each item gives one point where the scoring answer is “yes” for some questions and “no” for others. A systematic review and meta-analysis on the recommended GDS-15 cut-off score of ≥ 5 found a pooled sensitivity of 0.89 and specificity of 0.77. The samples included in the studies had an average age ranging from 66 to 87, and the majority of them excluded individuals with impaired cognition [5]. Conradsson et al. found that among very old people with Mini-Mental State Examination (MMSE) scores ≥ 10, the GDS-15 was useful for assessing depressive symptoms [6]. A meta-analysis found evidence that the scale's factor structure varies depending on linguistic and cultural factors, and the number of factors ranged from two to nine. [7].

The Swedish version of the GDS-15, translated in 1995, has only undergone partial validation, and there is still a need for comprehensive validation of the entire scale. In one study of the original 30-item version of the GDS that involved stroke patients, the GDS was compared with six other depression rating scales; however, the remaining scales are not commonly used in clinical practice in Sweden today. It showed Pearson correlation coefficients ranging from 0.37 to 0.88 for concurrent validity when comparing GDS to the other scales and 0.75 compared to a clinical measurement of depression severity [8]. A rare study of the Swedish version of the GDS-15 in older adults (aged 75.7 ± 6.1) found a high sensitivity of 94% and specificity of 88% for the cut-off value of ≥ 6 [9] to detect a major depressive episode in 17 of 113 volunteers, but minor depression was not investigated. Further, the test–retest reliability has not been tested in the Swedish version of the GDS-15.

There is a lack of studies among very old adults; one exception is Zhang et al. [10]. While there have been studies investigating the test–retest reliability of the GDS-15 [11, 12] to the best of the authors' knowledge, there is a lack of research utilizing these results to calculate the least significant change.

### Aim

This study aims to evaluate the validity and reliability of the Swedish version of the GDS-15 among very old adults ≥ 85 years. This study also examines whether there are differences in the scale depending on subgroups divided by sex, age group, and MMSE scores.

## Material and methods

### Data source

The sample used in this study was taken from the Umeå85 + /GErontological Regional DAtabase (GERDA) study. This study invited to participate, every second 85-year-old, randomly selected by their odd or even position in the population registry, every 90-year-old, and every 95-year-old or older. Participants were recruited from Umeå, an urban municipality in northern Sweden, and five rural municipalities in the county of Västerbotten. The study started recruiting during 2000–2002 and then every five years until 2017. After five years, previous participants were invited to participate again. In addition, new participants were recruited from the same area. The participants were included irrespective of ongoing treatment with antidepressants.

A convenience sample was collected in 2022 to be used for the test–retest reliability. Individuals from senior citizen organizations, stroke inpatients and outpatients as well as individuals in nursing homes who were above the age of 70 years and lived in the urban municipality of Luleå in the county of Norrbotten were invited to participate.

### Participants

Most participants in the Umeå85 + /GERDA study performed the GDS. Between 2000 and 2002, those with high GDS scores received a new visit within a few days by a physician specialist in geriatric medicine for a depression assessment, which included the Montgomery-Åsberg Depression Rating Scale (MADRS). However, other interviewers who were specifically trained for the task, such as medical students, nurses, or physiotherapists, could have performed the initial GDS assessments, and any ambiguities were settled by senior researchers. Although the scales were conducted on different days, possibly by different interviewers, and the participants were selected based on previous results, it was decided that all 104 participants from 2000–2002 would be included in the present study. This decision considered that the scales were performed within a few days of each other. In 2005, it was decided that the MADRS would be administered by all interviewers who were physicians or medical students who had completed their psychiatric clinical practice and were trained to use the scale. This meant that the MADRS and GDS assessments were performed at the same time by the same interviewer. Between 2000 and 2017, 418 assessments were conducted using the GDS and MADRS. Conradsson et al. [6] showed that GDS-15 scores in individuals who scored 10 or more on the MMSE were valid. Therefore, we removed those with an MMSE score below ten (18 assessments) and those with more than one unanswered GDS item (13 assessments). The remaining 387 assessments constituted the final sample and included 334 individual participants, of whom 46 participated more than once. The assessments were counted as individuals since the time between participation was five years or more.

All participants in the convenience sample were recruited in 2022 and assessed twice by the same author (JN), an experienced geriatrician.

### Assessments

The Montgomery-Åsberg Depression Rating Scale (MADRS) was designed in 1978 to be particularly sensitive to changes in depression during treatment [13]. The MADRS includes 10 items, and the version used in this study scored from 0 to 60 points, with higher scores indicating more depressive mood. Kyle et al. used a cut-off score ≤ 12 on the MADRS as "marked recovery" from depression when comparing two antidepressants in elderly depressed patients [14]. However, no consensus has emerged for a specific cut-off score for depression or remission, with scores varying from 4–12 [15]. The scale has shown good reliability and validity, with a sensitivity of 0.80 and specificity of 0.82 among individuals with a mean age of 81 and MMSE scores ≥ 20 points [16]. It is used in Swedish healthcare today to diagnose and detect persons with probable depression due to its close association with DSM-V depression criteria and to follow up on antidepressant treatment.

Cognition was assessed using the frequently used Mini-Mental State Examination (MMSE), which gives a rough estimate of various cognitive functions. The result is stated in points, with 30 as the maximum score. It is often used to express the degree of cognitive impairment, where 18–23 indicates mild impairment and ≤ 17 indicates severe impairment [17].

Cognitive assessment in the convenience sample was made with the Six Item Screener, which is suitable for telephone assessment. The Six Item Screener was chosen since some interviews were conducted over the telephone due to the COVID-19 pandemic. The Six Item Screener is a brief cognitive screening tool for identifying subjects with cognitive impairment. Each item can score one or zero points, and scores below four indicate cognitive impairment, with a sensitivity of 88.7% and specificity of 88.0% [18].

Activities of daily living (ADL) were assessed using the Barthel Index, where 20 points correspond to total independence and 0 points correspond to total dependence [19]. Participants living in nursing homes, including residential homes, nursing homes, and group dwellings for people with dementia disorders, were included.

## Statistics

### Concurrent validity

Concurrent validity between the GDS and the MADRS was measured using correlation calculations. Spearman's correlation was chosen as the analysis method after a graphical examination, which showed that the data were not normally distributed. A correlation was also examined between the individual items of the GDS and the MADRS.

### Cut-off (ROC analysis)

A scatter plot was used to visualize GDS-15 and MADRS scores. As part of our evaluation of the cut-off on GDS, we first needed to determine whether participants were depressed. For this purpose, we compared the MADRS with the DSM-V criteria for depression. The DSM-V requires five or more symptoms where at least one of the symptoms should be either a depressed mood or loss of interest. The symptoms of depressed mood and loss of interest are assessed in MADRS items 1, 2 (depressed mood), and 8 (loss of interest or pleasure). It was decided that participants with two points or more on four MADRS items, including either depressed mood or loss of interest, were considered to have major depression. However, items 6, "concentration difficulties", and 7, "lassitude", were counted as one symptom since they together were considered to assess the DSM-V criterion "diminished ability to think or concentrate, or indecisiveness". We chose four symptoms instead of five, considering that the DSM-V symptom "fatigue or loss of energy nearly every day" is not included in any MADRS item but can be assumed to be highly prevalent in the geriatric population. Participants who scored 2 points or more on one of the MADRS items 1, 2, and 8 but did not meet the criteria of the four symptoms described above were considered to have minor depression.

We used Fisher's r-to-z transformation test, a two-tailed test for independent samples, to compare the correlation coefficients across age groups, sex, and cognition subgroups.

T-tests and Chi-2 tests were conducted to detect significant differences in GDS, MADRS, MMSE, age, and sex between the groups enrolled in the study before or after 2005 since assessments with MADRS between 2000–2002 were made on indication GDS ≥ 5.

We used the Area Under the Curve (AUC) of the Receiver Operating Characteristics (ROC) curve to measure the performance of the GDS. The ROC curve was also used to assess the cut-off value with the highest specificity and sensitivity. AUC and ROC curves for the independent subgroups (sex, age group, and MMSE) were compared to identify significant differences in scale function.

### Construct validity (factor analysis)

Factor structure was computed through exploratory factor analysis using principal component analysis. The number of factors was determined using Kaiser's eigenvalue-greater-than-one rule and Cattell's scree plot. Factor loadings were redistributed with direct oblimin rotation to determine which items measure which factors.

### Internal reliability

Internal reliability, or consistency, demonstrates whether items of a scale measure the same construct and was analyzed by calculating Cronbach's alpha. Item-total correlation evaluates how an item correlates with the scale's total score. A correlation less than 0.2 indicates that the item might measure something other than the scale as a whole [20]. The scale was also tested to see if alpha increased when an item was removed, which is used to validate the items.

### Test–retest

Test–retest reliability was analyzed with correlation, Cohen’s weighted kappa, and Intraclass Correlation Coefficients (ICC). Cohen’s Kappa was deemed according to the following criteria: moderate (0.40–0.59), substantial (0.60–0.79), and outstanding (> 0.80) [21]. Absolute reliability or ICC was deemed according to the following criteria: poor (< 0.5), moderate (0.5–0.75), good (0.75–0.9), and excellent (> 0.9) [22]. The within-subject standard deviation or within-people mean square residual was calculated using ANOVA (the F-test in SPSS’s Scales module was used). The least significant change between the two tests was calculated using within-subject standard deviation multiplied by the $\sqrt{2}$ and 1.96, the latter to obtain the 95% confidence interval [23, 24, 25]. The least significant change is the minimum score needed to exceed a measurement error for a scale.

All analyses were performed using SPSS. IBM Corp. Released 2020. IBM SPSS Statistics for Windows, Version 27.0. Armonk, NY: IBM Corp. A two-tailed probability value of ≤ 0.05 was considered significant.

## Results

### Sample

Table 1 shows that the main sample consisted of 387 individuals with a mean age of 91.0 (± 5.0) years, 65.1% were women, 36.2% were living in nursing homes and 82.4% were living alone. The average number of years in school was 6.7 ± 2.2 and they had an MMSE score of 22.5 (± 5.2). The mean GDS score was 4.0 (± 3.0), 158 individuals (40.8% of the total) had a GDS score ≥ 5 points and the mean MADRS score was 5.0 (± 5.0). The convenience sample consisted of 60 individuals with a mean age of 80.7 (± 5.4) years, 40% were women, 20% were living in nursing homes and 46.7% were living alone. The average number of years in school was 10.5 ± 3.5 and the average Six Item Screener was 4.4 (± 1.6). The mean GDS was 3.2 (± 3.4) for the first assessment and 3.3 (± 3.4) for the second assessment. On the first assessment, there were 13 individuals (21.7%) who had a GDS score ≥ 5 points and on the second assessment 14 (23.3%).

**Table 1:** Basic characteristics of the main sample (*N* = 387) and convenience sample (*N* = 60)

Main sample		Convenience sample
Variable	*n* (%)	*n* (%)
Sociodemographic		
Female	252 (65.1%)	24 (40%)
Age^a^	90.7 ± 4.7 (85–103)	80.7 ± 5.4 (70–96)
Age groups		
85	110 (28.4%)	-
90	134 (34.6%)	-
≥ 95	143 (37.0%)	-
Number of years in school^a^	6.7 ± 2.2 (0–20)	10.5 ± 3.5 (7–19)
0–5 years	43 (12.0%)	0
6–7 years	241 (67.5%)	17 (28.3%)
8–9 years	42 (11.8%)	16 (26.7%)
≥ 10 years	31 (8.7%)	27 (45.0%)
Living in a nursing home	132 (36.2%)	12 (20%)
Living alone	299 (82.4%)	28 (46.7%)
Assessments		
MMSE^a^	22.5 ± 5.2 (10.0–30.0)	-
Barthel ADL-index^a^	17.0 ± 4.0 (0–20.0)	-
GDS-15^a^ First assessment	4.0 ± 3.0 (0–13.0)	3.2 ± 3.4 (0–15)
GDS-15^a^ Second assessment	-	3.3 ± 3.4 (0–15)
Days between GDS assessment		2.2 ± 1.5 (1–8)
MADRS^a^	5.0 ± 5.0 (0–28.0)	-
Six Item screener (points)^a,b^	-	4.4 ± 1.6 (0–6)
Health related		
Experienced pain past week	177 (61.9%)	-
Stroke history	58 (15.0%)	-
Sleep disorders	132 (46.2%)	-
Dementia	120 (31.5%)	-
Use of antidepressant drugs	60 (15.5%)	-

*SD* Standard Deviation, *MMSE* Mini-Mental State Examination, *ADL* Activities of Daily Living, *GDS* Geriatric Depression Scale, *MADRS* Montgomery-Åsberg Depression Rating Scale

^a^mean ± *SD* (Range)

^b^Six Item Screener: 0 – 6 points where less than four points indicate possible cognitive impairment. Suitable for telephone interview during Covid-19 pandemic

We considered the main sample of 387 assessments to be different individuals since they had been conducted at least five years apart. There were 46 participants in the main sample with two different assessments five years apart and removal of one of these yielded similar results for the validity calculations (data not shown). The change in Geriatric Depression Scale for these 46 participants is displayed in additional Fig. 1.

**Fig. 1:**
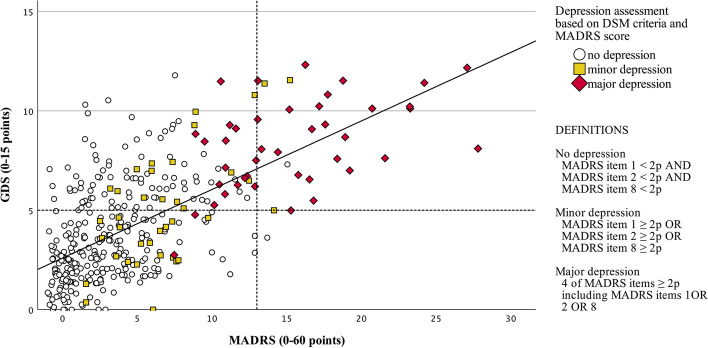
Scatter plot visualizing the main sample participants (*N* = 387) based on their GDS-15 and MADRS assessments. LEGEND: GDS = Geriatric Depression Scale. MADRS = Montgomery-Åsberg Depression Rating Scale. DSM = Diagnostic and Statistical. Manual of Mental Disorders. Solid line = regression (*R*^2^ linear = 0.411). Horizontal line = recommended GDS cut-off ≥ 5 for any depression. Vertical dotted line = suggested MADRS cut-off  ≥ 13 points for any depression, commonly used in Sweden

### Concurrent validity

Spearman's correlation between the total GDS and MADRS scores showed a correlation coefficient of 0.60, with the results presented in Table 2. Every item in the GDS showed a significant correlation with the total score on the MADRS; the coefficients ranged from 0.11–0.40, except for GDS item 9 ("Do you prefer to stay at home, rather than going out and doing new things?"). In addition, item 9 did not significantly correlate with any of the individual items within the MADRS.

**Table 2:** Spearman's correlation between MADRS and GDS-15 items and total score correlation in the main sample (*N* = 387)

	GDS1	GDS2	GDS3	GDS4	GDS5	GDS6	GDS7	GDS8	GDS9	GDS10	GDS11	GDS12	GDS13	GDS14	GDS15	GDS Total
MADRS1 "Apparent sadness"	.238^b^	.206^b^	.275^b^	.283^b^	.354^b^	0.079	.391^b^	.334^b^	0.059	0.099	.334^b^	.365^b^	.225^b^	.395^b^	.190^b^	.515^b^
MADRS2 "Reported sadness"	.338^b^	.176^b^	.362^b^	.272^b^	.400^b^	0.08	.380^b^	.339^b^	-0.02	0.033	.368^b^	.362^b^	.225^b^	.353^b^	.123^a^	.508^b^
MADRS3 "Inner tension"	.165^b^	.109^a^	0.083	0.04	.265^b^	.173^b^	.181^b^	.128^a^	0.027	0.057	0.054	0.082	0.048	0.058	0.09	.230^b^
MADRS4 "Reduced sleep"	.112^a^	.127^a^	.128^a^	0.06	0.071	0.037	.117^a^	.160^b^	0.018	-0.027	.181^b^	0.035	.120^a^	.109^a^	.105^a^	.199^b^
MADRS5 "Reduced appetite"	.115^a^	.173^b^	.210^b^	.134^b^	.262^b^	.106^a^	.246^b^	.256^b^	0.031	0.012	.329^b^	.260^b^	.171^b^	.244^b^	.114^a^	.364^b^
MADRS6 "Concentration difficulties"	.147^b^	0.09	.124^a^	0.06	.202^b^	0.045	.178^b^	.187^b^	0.056	.139^b^	.149^b^	.186^b^	.128^a^	.215^b^	.182^b^	.276^b^
MADRS7 "Lassitude"	.166^b^	0.054	.186^b^	.180^b^	.173^b^	0.031	.137^b^	.287^b^	0.073	0.06	.154^b^	.242^b^	.241^b^	.289^b^	.197^b^	.339^b^
MADRS8 "Inability to feel"	.113^a^	.151^b^	.183^b^	.149^b^	.215^b^	0.058	.257^b^	.103^a^	0.021	0.056	.197^b^	.172^b^	.168^b^	.179^b^	0.069	.292^b^
MADRS9 "Pessimistic thoughts"	.211^b^	.132^b^	.213^b^	.282^b^	.246^b^	.160^b^	.247^b^	.198^b^	0.062	0.039	.266^b^	.366^b^	.194^b^	.291^b^	.140^b^	.419^b^
MADRS10 "Suicidal thoughts"	.190^b^	.190^b^	.300^b^	.194^b^	.248^b^	0.05	.347^b^	.267^b^	-0.01	0.047	.326^b^	.370^b^	.257^b^	.380^b^	0.056	.450^b^
MADRS Total	.274^b^	.272^b^	.316^b^	.251^b^	.335^b^	.162^b^	.381^b^	.357^b^	0.056	.105^a^	.350^b^	.399^b^	.312^b^	.370^b^	.216^b^	.603^b^

*GDS* Geriatric Depression Scale, *MADRS* Montgomery-Åsberg Depression Rating Scale

^a^Correlation is significant at the 0.05 level (2-tailed)

^b^Correlation is significant at the 0.01 level (2-tailed)

According to the DSM-V, a diagnosis of depression requires the presence of either a depressed mood or a loss of interest. These symptoms are met by MADRS items 1, 2, and 8. Correlation analysis of these items with the total GDS score gave results of 0.52, 0.51, and 0.29, respectively. GDS items that strongly related to these MADRS items were 5 ("Are you in good spirits most of the time?") and 7 ("Do you feel happy most of the time? "), with coefficients ranging from 0.10–0.40.

Based on sex, age group (85, 90, and ≥ 95 years), and MMSE score (10–17, 18–23, and 24–30 points), no significant differences in correlation between GDS and MADRS were shown in any of the groups (data not shown).

### Cut-off (ROC analysis)

**Fig. 2:**
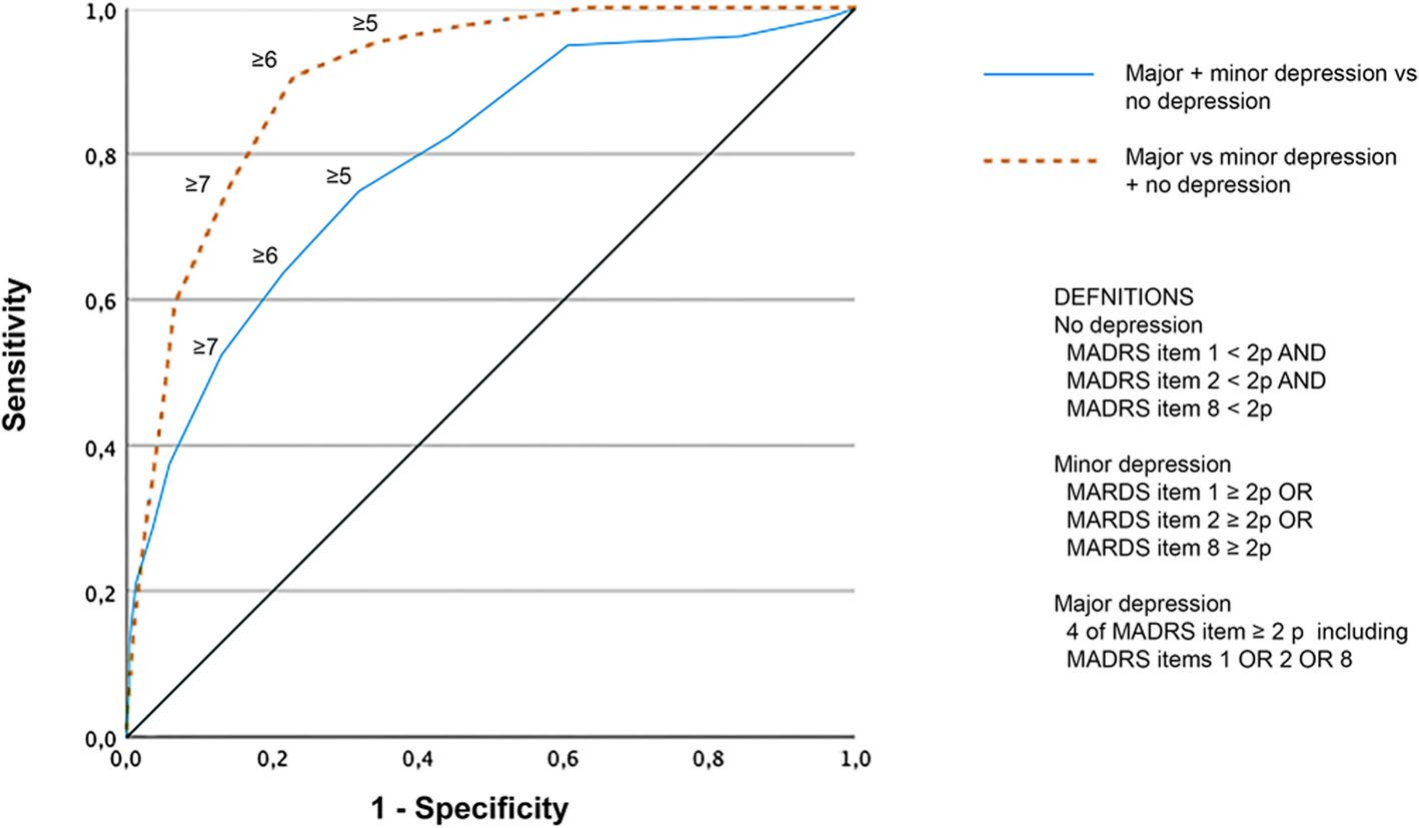
Receiver Operating Characteristic (ROC) curve for the main sample (*N* = 387)

Figure 1 shows a scatter plot visualizing the main sample participants based on their GDS-15 and MADRS assessments. Major depression, based on the criteria in this study, was distinguished in the area under the curve at a level of 0.90 (see Fig. 2). The recommended cut-off value of ≥ 5 resulted in a sensitivity of 95.2% and a specificity of 65.8%. The positive predictive value (PPV) was 25.3%, and the negative predictive value (NPV) was 99.1%. The sensitivity was 90.5% for the cut-off value ≥ 6, and the specificity was 77.1%. When distinguishing any depression (major and minor) from no depression, a cut-off ≥ 5 showed a sensitivity of 75.0%, specificity of 68.1%, PPV of 38.0%, and NPV of 91.3%. Cut-off ≥ 6 had a sensitivity of 63.8% and a specificity of 78.5%.

The sensitivity, specificity, PPV, and NPV for the individual subgroups (sex, age group, and MMSE scores) are shown in Table 3. The comparison of AUC for the subgroups showed no significant difference (data not shown).

**Table 3:** Sensitivity, specificity, PPV, and NPV for recommended cut-off ≥ 5 when GDS was compared to depression assessment based on DSM-V criteria and MADRS score (*N* = 387)

Group	Major depression (*n* = 42) vs. Minor + No depression (*n* = 345)				Major + Minor depression (*n* = 80) vs. No depression (*n* = 307)			
	Sensitivity %	Specificity %	PPV %	NPV %	Sensitivity %	Specificity %	PPV %	NPV %
All	95.2	65.8	25.3	99.1	75.0	68.1	38.0	91.3
Sex								
Male	92.3	72.1	26.1	98.9	71.4	72.8	32.6	93.3
Female	96.6	62.3	25.5	99.3	76.3	65.3	40.2	90.0
Age group								
85	87.5	68.6	18.0	98.6	62.5	69.2	26.6	91.6
90	100.0	67.0	29.1	100.0	89.7	72.4	47.3	96.2
≥ 95	94.4	62.4	26.6	98.7	68.6	63.0	37.5	86.1
MMSE								
10 – 17	100.0	54.8	34.9	100.0	78.8	61.4	60.5	79.4
18 – 23	88.9	62.6	30.2	96.9	67.9	61.8	35.9	85.9
24 – 30	100.0	71.2	14.5	100.0	79.0	73.0	24.2	97.0

Number of individuals with depression according to GDS-15 was 158 of 387

*PPV* Positive predictive value, *NPV* Negative predictive value

### Construct validity (factor analysis)

The Principal Component Analysis resulted in a Kaiser's eigenvalue over 1 for four factors, with a cumulative variance of 46.8%. Based on Cattell's scree plot, only one factor was validated as significant with a clear "elbow" in the graph. This factor alone accounted for as much as 23.5% of the variance, unlike the other three factors, which only explained between 7.1 and 8.7% separately (data not shown).

### Internal reliability

Internal reliability was evaluated with Cronbach's alpha for the main sample; the results are shown in Table 4. Cronbach's alpha for the total scale was 0.73 and corrected item-to-total correlations ranged from 0.07 – 0.49. Items 6, 9, and 10 correlated below 0.2, and removing these items yielded a higher alpha for the total scale. Removal of item 9 caused the highest increase in alpha to 0.74.

**Table 4:** Internal Reliability Statistics in the main sample (*N* = 387) and test–retest reliability in the convenience sample (*N* = 60)

Main sample			Convenience sample	
Item-Total Statistics			Test–retest reliability	
Cronbach's Alpha for the whole Scale	0.727		Assessment 1: 0.851 and assessment 2: 0.854	
	Cronbach's Alpha	Corrected Item-	Cohens kappa_w_	Absolute reliability (ICC)
	if Item Deleted	Total Correlation		
For the whole scale			0.71	0.95
GDS1 "Are you basically satisfied with your life?"	0.709	0.394	1.0	1.0
GDS2 "Have you dropped many of your activities and interests?"	0.720	0.284	0.62	0.76
GDS3 "Do you feel that your life is empty?"	0.697	0.459	0.66	0.82
GDS4 "Do you often get bored?"	0.707	0.392	0.52	0.69
GDS5 "Are you in good spirits most of the time?"	0.711	0.363	0.91	0.96
GDS6 "Are you afraid that something bad is going to happen to you?"	**0.729**	**0.186**	0.49	0.66
GDS7 "Do you feel happy most of the time?"	0.701	0.454	0.69	0.82
GDS8 "Do you often feel helpless?"	0.700	0.442	0.57	0.73
GDS9 "Do you prefer to stay at home rather than going out and doing new things?"	**0.740**	**0.070**	0.47	0.64
GDS10 "Do you feel you have more problems with memory than most?"	**0.735**	**0.118**	0.62	0.77
GDS11 "Do you think it is wonderful to be alive now?"	0.710	0.394	0.70	0.83
GDS12 "Do you feel pretty worthless the way you are now?"	0.697	0.456	0.69	0.82
GDS13 "Do you feel full of energy?"	0.721	0.281	0.79	0.89
GDS14 "Do you feel that your situation is hopeless?"	0.698	0.494	0.57	0.73
GDS15 "Do you think that most people are better off than you are?"	0.719	0.274	0.66	0.80

For test–retest reliability: There was linear heteroscedasticity, however when removing individuals scoring zero there was no linear heteroscedasticity. There was only one rater for the Convenience test–retest reliability sample (JN)

*GDS* Geriatric Depression Scale, Cohens kappa_w_ weighted kappa, *ICC* Intraclass Correlation Coefficients

### Test–retest

Table 4 shows that Cohen’s weighted kappa was 0.71 for the convenience sample, and item kappa varied between 1.0–0.47. The ICC was 0.95 for the whole sample, and the item ICC ranged between 1.0 and 0.64. The item with the lowest kappa and ICC was item 9. The within-people residual mean square was 1.08, and the least significant change was calculated to be 2.99 with a 95% confidence interval.

## Discussion

The study has demonstrated good validity and internal reliability of the Swedish version of the GDS-15 among very old adults regardless of sex, age group, or MMSE scores ≥ 10. The scale showed high values for sensitivity and specificity, 95.2% and 65.9%, respectively, when compared to a depression assessment based on MADRS used according to DSM-V. We believe the results were comparable with other studies in this field and find that the Swedish version of GDS-15 is suitable as a screening tool for depression among very old people.

### Concurrent validity

Concurrent validity was examined using the Montgomery-Åsberg Depression Rating Scale (MADRS), which has previously demonstrated good validity in measuring depression among individuals with a mean age of 81 and Mini-Mental State Examination (MMSE) scores ≥ 20 points [14]. Spearman's correlation between the two scales showed an acceptable correlation, indicating that the Swedish version of the GDS is also a valid screening tool for depression among the very old.

In the correlation analysis between the total GDS score and MADRS items 1, 2 (depressed mood), and 8 (loss of interest), it appears that the overall GDS scale captures a form of depression characterized by a greater emphasis on "sadness" rather than a focus on "loss of interest". It is also possible that very old adults give up their interests for reasons other than depression and, therefore, they less frequently experience this particular depressive pattern.

### Cut-off (ROC curve)

We created a new variable for major and minor depression by utilizing the MADRS to meet the criteria outlined in the DSM-V. We believe this yielded a superior result compared to using a cut-off score for MADRS, since there is no consensus on which cut-off to use for MADRS and, further, DSM-V is often used for diagnosing depression. Additionally, assessments were performed by a physician or trained medical student rather than the participants themselves, and almost all DSM criteria are found in the MADRS.

As shown in Fig. 1, the GDS cut-off ≥ 5 misses very few individuals with major depression but presents some difficulty for those with minor depression. The scatter plot is comparable to a Canadian study comparing GDS with MADRS [26] in a younger sample with a mean age of 75 ± 6.5 years. It can be argued that a lower cut-off would be better for screening since it increases sensitivity and thus further reduces the risk of missing individuals with depression, which was supported by de Craen et al. [27], who argued that a cut-off of ≥ 4 or ≥ 3 would be better when screening for depression. Nevertheless, the sensitivity for the generally recommended cut-off ≥ 5 is so high that a lower cut-off would not increase sensitivity sufficiently compared to the decrease in specificity that this entails. Thus, we argue that the generally recommended cut-off ≥ 5 is also reasonable for the Swedish translation of the GDS-15 when used to screen for both major and minor depression.

### Construct validity (factor analysis)

The four-factor model proposed by Kaiser's eigenvalue resulted in factors that were too similar, making them unsuitable for use as distinct factors. Therefore, we propose a one-factor model, as derived from Cattel's Scree plot, which clearly speaks for a one-factor model, and we named that factor "depression". This is comparable with a Chinese study that found a two-factor model according to Kaiser's Eigenvalue. However, factor number two was challenging to interpret and not considered meaningful, and Cattel's Scree plot showed a one-factor model [28].

### Internal reliability

The Cronbach's α of 0.73 is comparable to one Swedish GDS-15 study with a Cronbach's α ranging from 0.636 to 0.775, depending on MMSE scores [6]. Other studies have found alpha scores ranging from 0.55 for the Dutch translation [29] to 0.90 for the Iranian translation [30]. The item-total correlation below 0.2 for items 6, 9, and 10 and the increase in alpha when removed may indicate that the items measure something different from the scale as a whole.

GDS item 9 ("Do you prefer to stay at home rather than going out and doing new things?") has, in previous studies, shown low results for item 9 in various analyses [31, 32], indicating that this item measures something other than depression or that it is just not relevant among very old adults. Item 10 ("Do you feel you have more problems with your memory than most?") also showed poor results. An item response theory (IRT) analysis of the Swedish GDS-15 identified item 10 with the highest difficulty, indicating that the item marks an exceptionally high degree of depression [31]. This could explain the low results in this study, as very high GDS scores were rare.

### Test–retest

The results show that Cohen’s weighted kappa was substantial, and the ICC was excellent for the GDS-15. Little is known about how sensitive the GDS-15 is to change. The least significant change between two measurements in this study was 2.99 tested with a mean of two days apart, i.e., there must be at least three points between two tests to exceed measurement error on an individual level. However, the clinically relevant change between the two measurements might be larger. On average, two days between testing in this study seems suitable to detect measurement errors and not change in mood.

### Strengths and weaknesses

Few studies on GDS have been performed among very old adults. However, we know that depression becomes more common with age, and this age group will benefit significantly from having a well-functioning screening scale for depression. Therefore, a strength of this study is the high age of our study sample compared to previous studies [26, 32].

The large number of assessments included in the study is another strength.

A weakness of this study is that we compared GDS to a different assessment scale instead of a physician-established diagnosis of depression. However, MADRS was performed by trained professionals and compared to DSM-V criteria for a depression assessment, which is often the case in clinical settings. Additionally, the MADRS needs to be better studied among very old adults.

## Conclusion

The Swedish version of the GDS-15 showed good validity and internal reliability for screening for depression in very old adults, ≥ 85 years, with no difference regarding sex, age groups, or MMSE scores ≥ 10. The generally recommended cut-off value of ≥ 5 seems reasonable for use with the Swedish translation.

### Supplementary Information

**Supplementary Material 1.**

## Data Availability

The datasets generated and/or analysed during the current study are not publicly available due to General Data Protection Regulation (GDPR) and ethical approval used in this study but are available from the corresponding author on reasonable request.
