# Supplementary figures and images for: Validity and test–retest reliability of the Swedish version of the Geriatric Depression Scale among very old adults
Source: BMC Geriatr. 2024 Mar 18;24:261. doi: 10.1186/s12877-024-04869-7 (PMC10946128; doi:10.1186/s12877-024-04869-7)

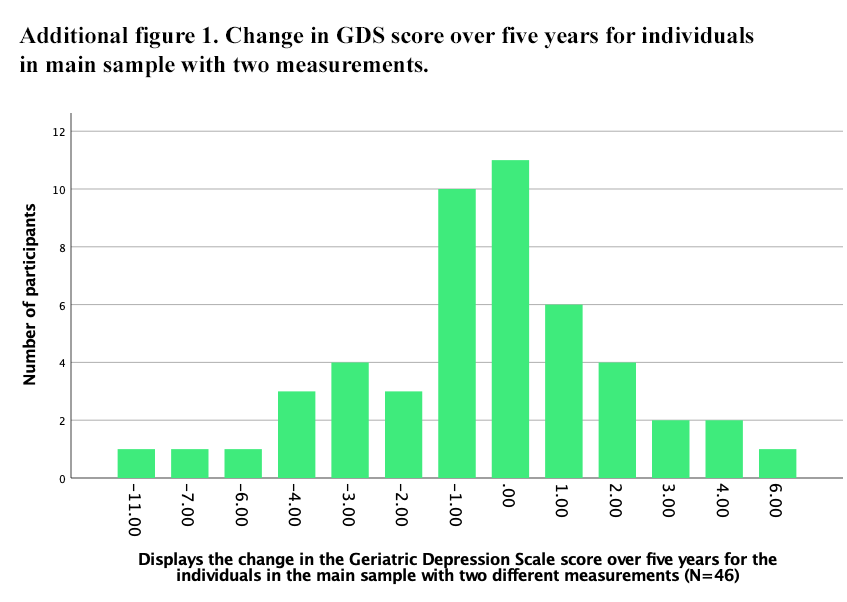

Supplement: Supplementary file 1 — Supplementary Material 1. [file 12877_2024_4869_MOESM1_ESM.png]
